# Retraction: Clinical efficacy of neurofacilitation technology combined with rehabilitation training based on cerebral blood flow velocity and cerebral metabolism in children with cerebral palsy

**DOI:** 10.3389/fmed.2026.1867338

**Published:** 2026-05-04

**Authors:** Frontiers Editorial Office

The journal retracts the 25 June 2025 article cited above.

Following publication, concerns were raised regarding the validity of the data in the article. The authors failed to provide the raw data or a satisfactory explanation during the investigation, which was conducted in accordance with Frontiers' policies. Given the concerns, and the lack of raw data, the editors no longer have confidence in the findings presented in the article.

This retraction was approved by the Chief Editors of Frontiers in Medicine and the Chief Executive Editor of Frontiers. The authors received a communication regarding the retraction and had a chance to respond. This communication has been recorded by the publisher.

